# Assessment of hemispheric dominance for receptive language in pediatric patients under sedation using magnetoencephalography

**DOI:** 10.3389/fnhum.2014.00657

**Published:** 2014-08-21

**Authors:** Roozbeh Rezaie, Shalini Narayana, Katherine Schiller, Liliya Birg, James W. Wheless, Frederick A. Boop, Andrew C. Papanicolaou

**Affiliations:** ^1^Department of Pediatrics, Division of Clinical Neurosciences, University of Tennessee Health Science CenterMemphis, TN, USA; ^2^Neuroscience Institute, Le Bonheur Children’s HospitalMemphis, TN, USA; ^3^Division of Pediatric Neurology, Department of Pediatrics, University of Tennessee Health Science CenterMemphis, TN, USA; ^4^Department of Neurosurgery, University of Tennessee Health Science CenterMemphis, TN, USA

**Keywords:** magnetoencephalography, non-invasive, language mapping, sedation, epilepsy, children

## Abstract

Non-invasive assessment of hemispheric dominance for receptive language using magnetoencephalography (MEG) is now a well-established procedure used across several epilepsy centers in the context of pre-surgical evaluation of children and adults while awake, alert and attentive. However, the utility of MEG for the same purpose, in cases of sedated patients, is contested. Establishment of the efficiency of MEG is especially important in the case of children who, for a number of reasons, must be assessed under sedation. Here we explored the efficacy of MEG language mapping under sedation through retrospective review of 95 consecutive pediatric patients, who underwent our receptive language test as part of routine clinical evaluation. Localization of receptive language cortex and subsequent determination of laterality was successfully completed in 78% (*n* = 36) and 55% (*n* = 27) of non-sedated and sedated patients, respectively. Moreover, the proportion of patients deemed left hemisphere dominant for receptive language did not differ between non-sedated and sedated patients, exceeding 90% in both groups. Considering the challenges associated with assessing brain function in pediatric patients, the success of passive MEG in the context of the cases reviewed in this study support the utility of this method in pre-surgical receptive language mapping.

## INTRODUCTION

Non-invasive assessment of hemispheric dominance for receptive language using magnetoencephalography (MEG) is now a well-established procedure used across several epilepsy centers in the context of pre-surgical evaluation of children and adults while awake, alert, and attentive. Specifically, localization of receptive language cortex and subsequent estimation of hemispheric dominance using MEG has most readily been achieved employing a recognition memory task for spoken words, based on hemispheric differences in the degree of activity in the temporo-parietal cortex (Breier et al., 1999; Papanicolaou et al., 2004, 2006). In particular, the reliability with which this protocol has been used to establish hemispheric dominance for receptive language in children has been shown in several normative, as well as clinical, cohorts. Moreover, the suitability of MEG language mapping protocols as an alternative to the Wada procedure have been addressed over the course of several validation studies, with concordance rates ranging from 87% in the study with largest sample to date (Papanicolaou et al., 2004) to 100% agreement in the first sub-sample of patients of the same series (Breier et al., 1999), with the rest of the studies reporting uniformly, high agreement (Breier et al., 2001; Maestú et al., 2002; Hirata et al., 2004; Bowyer et al., 2005; Merrifield et al., 2007; Doss et al., 2009; McDonald et al., 2009; Hirata et al., 2010; Findlay et al., 2012; Tanaka et al., 2013).

Nevertheless, establishment of the efficiency of MEG as a functional mapping tool is especially important in the case of children who, for a number of reasons (e.g., age; developmental delay; general anxiety), must be assessed under sedation, typically achieved through administration of one of the following agents: dexamedetomidine, etomidate, sevoflurane, midazolam, fentanyl, and more commonly- as is the case in our center- propofol (Szmuk et al., 2003; Balakrishnan et al., 2007; König et al., 2009). Given the observation by some that certain anesthetics may induce cerebral metabolic depression, and consequently affect cognitive function (Heinke and Schwarzbauer, 2002; Heinke et al., 2004), the feasibility of obtaining reliable brain activation patterns associated with higher cognitive processes, such as language, in sedated individuals requires further investigation, given the prominent role of MEG in pre-surgical functional mapping.

To date, the challenge of passively obtaining reliable language activation maps, under sedation, has been addressed in only a handful of studies, primarily using functional magnetic resonance imaging (fMRI). Specifically, it has been reported that children under propofol sedation exhibit patterns of left hemisphere activation in response to auditory linguistic stimuli comparable to those observed in non-sedated individuals (Souweidane et al., 1999; Gemma et al., 2009), with others reporting similar patterns among sedated and non-sedated children during song, as well as speech, perception (Lai et al., 2012). Furthermore, the utility of passive language mapping with MEG has also been demonstrated recently in small series of cases, with the observation that patients undergoing subsequent resective surgery for epilepsy show no evidence of postoperative language deficits (Van Poppel et al., 2012).

In this report, we addressed whether the cortical mechanisms of linguistic processing of speech stimuli are sufficiently activated under sedation to allow for the determination of hemispheric dominance for receptive language. Specifically, we explored the efficacy of MEG language mapping under sedation through retrospective review of 95 consecutive pediatric patients, who underwent our receptive language protocol as part of routine clinical evaluation. If administration of anesthetic agents indeed results in the suppression of language related activity, we hypothesized that up to half of patients assessed under sedation would exhibit a departure from the higher incidence of left hemisphere dominance for language expected in non-sedated patients, similar to that of the general population.

## MATERIALS AND METHODS

Ninety-five consecutive patients (46 non-sedated, 6–18 years of age; 49 sedated, 18 months–15 years of age) were identified through retrospective review of clinical evaluations performed at the Epilepsy Monitoring Unit of the Le Bonheur Comprehensive Epilepsy Program, Le Bonheur Children’s Hospital, who underwent functional brain mapping with MEG between July 2012 and December 2013. The study was approved as a retrospective chart review by the Institutional Review Board of the University of Tennessee Health Science Center. Detailed workup for each patient included: (1) medical history and physical examination; (2) structural brain evaluation (e.g., MRI); (3) continuous scalp video electroencephalography (V-EEG) monitoring, (4) interictal scalp EEG/MEG, and (5) neuropsychological evaluation. Importantly, in cases where patients were of an appropriate age and/or behavioral difficulties did not impede cooperation, neuropsychological assessment of verbal skills was achieved using the following instruments: (1) Peabody Picture Vocabulary Test – Fourth Edition – (PPVT-4) to assess receptive vocabulary skills; (2) Boston naming test (BNT) to assess confrontational word retrieval; (3) *Verbal fluency* subtest of the Delis-Kaplan Executive Functioning System (D-KEFS) to assess semantic and phonemic fluency; (4) *Story memory* and *verbal learning* subtests of the Wide Range Assessment of Memory and Learning- Second Edition – (WRAML-2) to assess contextual and non-contextual verbal memory. Functional brain mapping with MEG was performed for each patient at the request of the referring epileptologist for further clinical evaluation. In addition to patients referred for evaluation of seizure disorder, six patients included in this retrospective review were admitted through the Le Bonheur Children’s Hospital Neurosurgery Service for evaluation prior to a planned tumor resection, and two patients for planned resection of an arterio-venous malformation (AVM). These eight patients underwent the aforementioned workup, with the exception of video EEG and interictal scalp EEG/MEG. A summary of the clinical and demographic characteristics of all patients included in this review is presented in **Table 1**.

**Table 1:** Clinical and demographic characteristics of patients having undergone receptive language mapping with and without sedation.

	Non-sedated	Sedated
N	46	49
Gender	24 M/23 F	21 M/28 F
Age range (Mean ± SD)	6–18 years (12.8 ±3.4)	18 months–15 years (6.1 ±****3.2)
Handedness	37 Right/9 left	27 Right/15 left/7 undetermined
**Chief complaint**
*Symptomatic Partial Seizures*	17	18
*Symptomatic Generalized Seizures*	3	15
*Symptomatic Mixed Generalized Seizures*	–	6
*Cryptogenic Partial Seizures*	9	–
*Cryptogenic Mixed Seizures*	1	4
*Idiopathic Generalized Seizures*	2	–
*Idiopathic Mixed Generalized Seizures*	3	–
*Paroxysmal Events*	6	3
*Neuroepothelial Tumor*	1	–
*Ganglioglioma*	2	–
*Astrocytoma*	1	–
*Ependymoma*	–	1
*Cervical Medullary Tumor*	–	1
*Aterio-Venous Malformation (AVM)*	1	1

### PROCEDURES

#### Induction of anesthesia

Sedation was administered according to the recently described protocol for the administration of general anesthesia during MEG (Birg et al., 2013). Initially, patients underwent pre-anesthetic evaluation by an anesthesiologist, which consisted of a review of medical history, and identification of contraindications for sedation (such as respiratory illness, fever, high blood pressure, and heart rate <60 beats/min). Patients did not receive any premedication prior to induction of intravenous (IV) sedation. General anesthesia was induced by propofol injection. The sedation was maintained by a propofol infusion rate of 2–10 mg/kg/h. Patients received oxygen via a nasal cannula. A blood pressure cuff and a pulse oximeter probe were placed on a lower extremity, away from the MEG sensors. All patients maintained spontaneous respiration. During breaks in the MEG data acquisition, the anesthesiologist/nurse titrated the anesthetic drugs and monitored the patient from outside the magnetically shielded room (MSR) scanner. The anesthesia machine and monitoring equipment were placed outside the MSR and extended breathing circuits, IV lines, and monitoring equipment were passed through a porthole in the MSR wall to the patient. The patient’s EEG, electrocardiogram (ECG), arterial blood pressure, end-tidal CO_2_, and temperature were monitored throughout the MEG testing.

#### Brain activation tasks

Localization of language specific cortex and subsequent determination of hemispheric dominance for receptive language was adapted from the continuous auditory word recognition protocol previously described by Papanicolaou et al. (2004) in a large-scale studies detailing specific procedures for determining hemispheric dominance for language functions using MEG. Immediately prior to the commencement of the MEG scan, patients instructed to “try to remember” a set of five words, deemed as targets. Depending on the patient’s overall verbal memory capacity, the targets were presented once or twice. Subsequently, during the MEG scanning, the five target words were repeated in a different random order, mixed with a different set of 30 distractors (non-repeating words) in each of four blocks of stimuli. Stimuli were presented for 1 s, one at a time (with a randomly varied interstimulus interval of 2–3 s), and delivered binaurally via plastic tubes terminating in ear inserts at the patient’s outer ear. Target words (*jump, please, little, drink,* and *good*) included 4 monosyllabic and one disyllabic word, and had a mean frequency in the Zeno et al. (1995) G6-7 corpus of 158 occurrences per million (range: 32–194 occurrences). A slightly higher proportion of distractors were disyllabic (40%) and the remaining monosyllabic, with a mean frequency of occurrence of 150 words per million in the same corpus (range: 18–820). During the procedure, patients were asked to lift their index finger of the dominant hand whenever they detect a repeated (one of the five targets) word. In the case of sedated patients, this protocol was modified to conform to a passive task given the state of the patients. Specifically, while the target words were presented immediately prior to commencement of the MEG scan, as was the case in non-sedated patients, sedated patient’s overall verbal memory capacity was n ot assessed. Consequently, sedated patients were not required to respond to the occurrence of the target words, which excluded the possibility of assessment of their verbal memory performance.

#### Imaging procedures

Magnetoencephalography recordings were obtained with a whole-head neuromagnetometer array (4-D Neuroimaging, Magnes WH3600) equipped with 248 first-order magnetometer coils and housed in a magnetically shielded chamber. The position of the sensors relative to the patient’s head was determined using five coils, three of which were anchored to the fiduciary points (nasion, left, and right periauricular points) and two on the forehead. The coils were activated briefly by passing a small current through them, at the beginning and then again at the end, of the recording session and their precise location in three-dimensional space was determined using a localization algorithm native to the recording system software. During the same process, the patient’s head shape was digitized using a stylus for subsequent localization of activity sources. The magnetic flux measurements were digitized at 508 Hz, and filtered off-line with a band pass filter between 0.1 and 20 Hz, baseline corrected (150 ms pre-stimulus onset) to remove DC drifts, and subjected to a noise reduction algorithm that is part of the 4D-Neuroimaging software.

Structural MR images were obtained using either: (1) a sagittal T1-weighted 3D MPRAGE sequence acquired on a Siemens Verio scanner (Siemens AG, Munich, DE) equipped with a 32 channel head coil (176 slices, FOV 256 mm, voxel size 1.0 mm × 1.0 mm × 1.0 mm, *TE/TR* = 2.6/2530 ms, 512 × 512 matrix, Flip angle 7°); or (2) an axial T1-weighted 3D FSPGR sequence acquired on a GE Signa HDxt scanner (General Electric, Milwaukee, WI) equipped with an 8-channel head coil (220 slices, FOV 256 mm, voxel size 0.9 mm × 0.9 mm × 1.0 mm, *TE/TR* = 3.7/8 ms, 512 × 512 matrix, Flip angle 12°).

#### Data analysis procedures

Following filtering, single-trial MEG event-related field segments (ERFs) in response to 110–125 stimulus presentations were averaged and brain activity sources were modeled as single equivalent current dipoles (ECDs) and fitted independently at successive 2 ms intervals (Sarvas, 1987; Papanicolaou et al., 2004; Simos et al., 2005) using the 4D-Neuroimaging proprietary software. The algorithm searched for the source most likely to have produced the observed magnetic field distribution at a given point in time. For a given point in time, the ECD fitting algorithm was applied to the magnetic flux measurements obtained from a group of 34–38 magnetometers, always including both magnetic flux extrema. Source solutions were considered satisfactory if they were associated with a correlation coefficient of at least 0.9 between the observed and the “best” predicted magnetic field distribution, and occurred between 200–800 ms after stimulus onset to ensure activity sources represent language-related activity, rather than modality-specific sensory activation (Simos et al., 1998; Szymanski et al., 2001; Halgren et al., 2002; Helenius et al., 2002). The location of each estimated dipolar source was determined with reference to a Cartesian coordinate system based on three fiducial points on the head (the nasion and external meatus of each ear), and subsequently approximated by co-registering these points to the patient’s high-resolution anatomical MRI.

Receptive language cortex was identified by evoked activity sources computed during the late portion (>200 ms) of the ERF waveform, falling within fronto-temporal, temporo-parietal, and mesial temporal regions, for each hemisphere. Final laterality judgments for receptive language for MEG-derived activation maps were based on two criteria. First, a laterality index was calculated by comparing the number of acceptable late activity sources observed in the right and left hemispheric activation [*LI*= (RH - LH)/(RH + LH)], with a range from +1 to -1, with positive values indicating left hemispheric dominance and negative numbers indicating right hemispheric dominance. Index values between 0.1 and -0.1 were considered to be indicative of bilaterally symmetric activation. Second, determination of laterality also considered the spatial extent of activation, namely the degree to which dipolar sources associated with the late portion of the ERF waveform engaged association regions critical to supporting receptive language function.

## RESULTS

Successful completion of laterality assessment for receptive language was achieved in 78% (*n* = 36) and 55% (*n* = 27) of non-sedated and sedated patients, respectively (**Table 2**). Across both groups of patients, cases with data deemed unusable for analysis was associated with several sources of artifact, including vagus nerve stimulator, ventriculoperitoneal shunt, orthodontic devices, excess epileptiform discharges, and environmental noise interfering with ambient recording environment.

**Table 2:** Profiles of non-sedated and sedated patients included in final analysis and rationale for exclusion of cases not considered for laterality assessment.

	Non-sedated	Sedated
N	36/46 (78%)	27/49 (55%)
Gender	18 M/18 F	13 M/14 F
Age range (Mean ± SD)	6–18 years	18 months–11 years
Handedness	(13.2 ± 3.0) 32 Right/4 left	(5.3 ± 2.8) 15 R/6 L/5 undetermined
**Artifact source**
*VNS*	1	8
*VP Shunt*	–	1
*Orthodontic Devices*	2	1
*Excessive Epileptiform Activity*	2	–
*Environmental Noise (e.g., Intraoperative MRI; Medical monitoring devices)*	5	12

The number of total dipolar (activity) sources estimated between the two patient groups, across both hemispheres, did not significantly differ (mean ± SEM: 239 ± 28 in the sedated group vs. 244 ± 23 in non-sedated group, *p* = 0.85). Furthermore, a similar comparison at the hemispheric level revealed no significant differences between the two groups. On average (±SEM), the number of dipoles estimated in the left hemisphere was 287 ± 21 in the non-sedated group, and 273 ± 30 in the sedated group (*p* = 0.7), and in the right hemisphere it was 201 ± 23 in the non-sedated group, and 206 ± 24 in the sedated group (*p* = 0.9).

**FIGURE 1:**
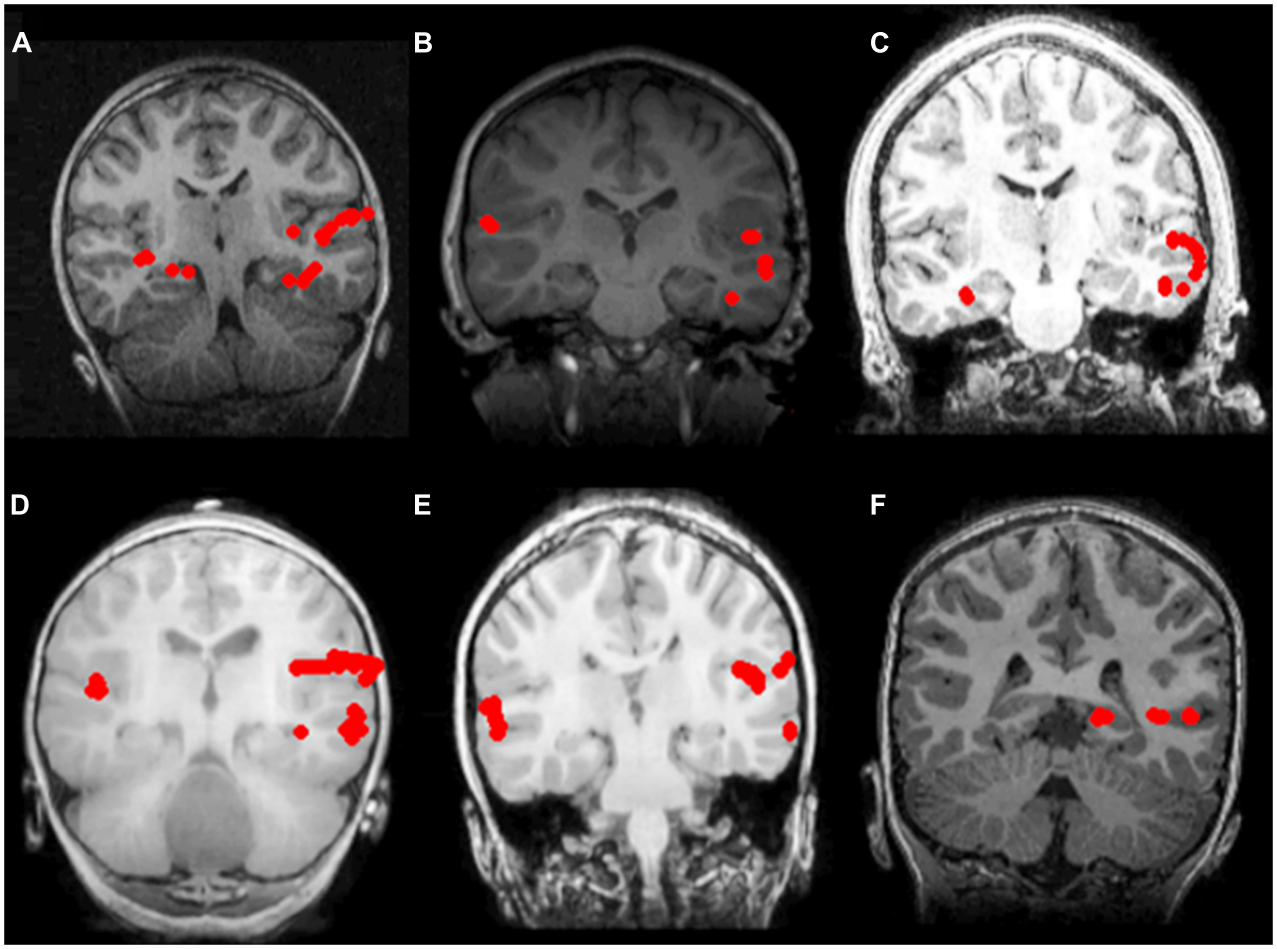
**Receptive language mapping with MEG with and without sedation.** Language activity (dipolar) sources are represented as solid red circles projected onto the patients’ MRI, displayed in radiological convention. Left hemispheric dominance for receptive language in patients evaluated without sedation **(A–C)**, and with sedation **(D–F)**. **(A)** 7 year-old female with symptomatic frontal lobe epilepsy; **(B)** 8 year-old female with ganglioglioma of the left temporal lobe; **(C)** 16 year-old male with symptomatic partial seizures of right temporal lobe origin; **(D)** 2 year-old male with cervical medullary tumor; **(E)** 6 year-old male with symptomatic epileptic spasms of right hemisphere origin; **(F)** 6 year-old male with symptomatic partial seizures of left temporal lobe origin.

As summarized in **Table 3**, there was no significant difference in the proportion of non-sedated (91.6%) and sedated (92.6%) patients deemed to be left hemisphere dominant for receptive language (Fisher’s exact test, *p* = 0.636). Moreover, among non-sedated and sedated individuals, two patients in each group were found to demonstrate bilateral representation for receptive language, with one patient in the non-sedated group also having been judged to be right hemisphere dominant. Characteristic brain activation profiles on the basis which laterality judgments for receptive language were made in non-sedated and sedate patients are displayed in **Figure 1**.

**Table 3:** Judgments on hemispheric dominance for receptive language in non-sedated and sedated patients.

	Non-sedated	Sedated
Left hemisphere	33	25
Right hemisphere	1	0
Bilateral	2	2

**FIGURE 2:**
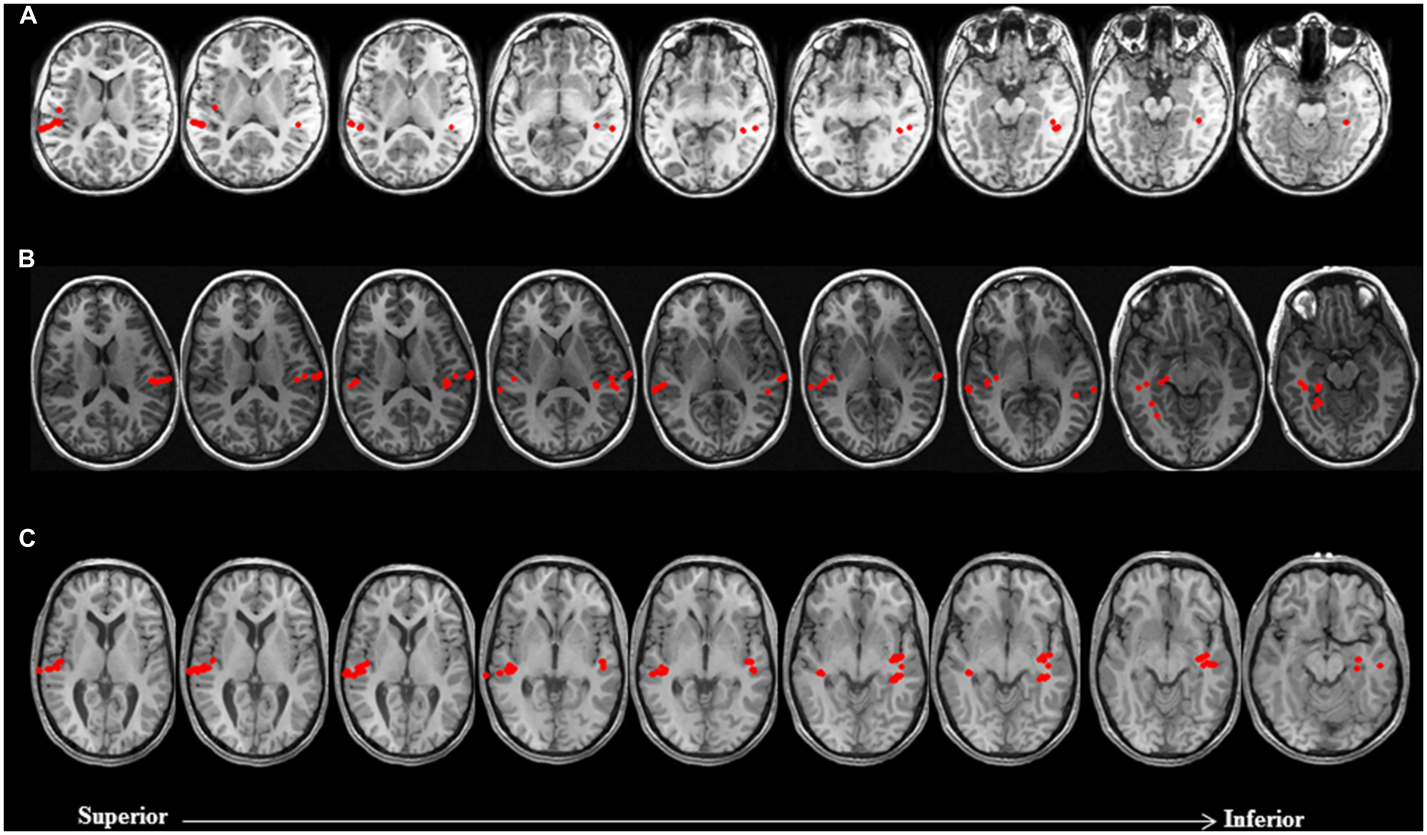
**Discordance between laterality judgments based on LI estimates and visual inspection of brain activation profiles in three non-sedated patients.** Language activity (dipolar) sources are represented as solid red circles projected onto the patients’ MRI, displayed in radiological convention. **(A)** 9 year-old female with symptomatic partial epilepsy of left temporal lobe origin was deemed to be right dominant for receptive language based on LI (-0.54). Visual inspection of brain activation profiles revealed despite a greater number of activity sources concentrated in the auditory cortex of the right hemisphere, posterior and mesial temporal engagement in the left hemisphere suggested that patient was left dominant for receptive language. **(B)** 14 year-old female with cryptogenic partial epilepsy of left temporal lobe origin was deemed to be left dominant for receptive language based on LI (0.43). Visual inspection of brain activation profiles revealed despite a greater number of activity sources concentrated in the auditory cortex of the left hemisphere, lateral and mesial temporal engagement in the right hemisphere suggested that patient was right dominant for receptive language. **(C)** 17 year-old male with idiopathic generalized seizures was deemed to have bilateral representation for receptive language based on LI (0). Visual inspection of brain activation profiles revealed despite an equal number of activity sources bilaterally, posterior middle and mesial temporal engagement in the left hemisphere suggested that patient was left dominant for receptive language.

As mentioned earlier, the approach taken for making clinical judgments regarding hemispheric dominance for receptive language took into account both the conventional quantitative LI, as well as visual inspection of brain activation profiles to assess the spatial extent of activity within the left and right hemispheres. On the basis of this approach, it is noteworthy that across both non-sedated and sedated groups, final laterality judgments in 26 out of the 63 patients with data deemed usable for analysis exhibited a discord between the traditional LI scores and qualitative assessment of brain activation maps. In particular, among 12 patients with an LI score suggestive of bilateral language representation, consideration of the spatial distribution of activity sources rendered final laterality judgments as left hemisphere dominant for receptive language. Moreover, in 11 patients judged to be right hemisphere dominant for receptive language according to LI scores, nine were deemed to be left hemisphere dominant and two as bilateral. Furthermore, of three patients with LI scores indicating left hemisphere dominance, one was deemed to be right dominant and two as bilateral. Examples of cases where hemispheric differences in the spatial distribution of activity sources, primarily characterized by clusters of activity outside the primary auditory cortex, that resulted in reconsideration of final laterality judgments based solely on LI scores, are provided in **Figure 2**.

## DISCUSSION

In the present study, we evaluated the efficiency of MEG in establishing hemispheric dominance for receptive language in pediatric patients under propofol sedation, compared to patients assessed without sedation. Localization of receptive language cortex and subsequent determination of laterality was successfully achieved in 78 and 55% of non-sedated and sedated patients, respectively. While cases excluded from analysis were affected by similar sources of mechanical and/or biological artifact in both groups of patients, this was found to be more frequent in sedated patients, thus leading to smaller proportion of successful laterality assessments in this group. However, the proportion of patients deemed left hemisphere dominant for receptive language did not differ between non-sedated and sedated patients, exceeding 90% in both groups, overlapping with the accepted rate of incidence of left-lateralized individuals in the general population (Moser et al., 2011).

The relatively high rate of successful laterality assessments in both non-sedated and sedated patients, as well as similarities in the lateralization estimates between the two groups, make a strong case for the adoption of the passive receptive language mapping protocol discussed in the current study. For example, in cases where surgical intervention is warranted, either for treatment of intractable seizures or tumor resection, passive language mapping with MEG may be a suitable alternative to the Wada test and direct cortical stimulation mapping which, from a practical perspective, may be difficult to carry out in pediatric patients due to either age or inability to tolerate the demands posed by these procedures. Indeed, considering that in the present study sedation was administered to mitigate behavioral difficulties associated with developmental delay in a large number of patients, passive language mapping may in some cases better characterize the organization of linguistic processes, as opposed to more conventional language assessments that rely on patient cooperation. Nevertheless, in a broader context of the goal of the present study, it is worth noting that in patients where formal neuropsychological testing is feasible, performance on independent measures of linguistic ability may be accounted for by language profiles derived using MEG, as well as other neuroimaging modalities. For example, the capacity for verbal memory and receptive vocabulary skills assessed behaviorally may in fact reflect the spatiotemporal profile of receptive language function obtained using the present MEG protocol. However, despite these assessments being routine clinical practice in our center, the unavailability of complete neuropsychological data in the course of the chart review presented in this study made such a comparison difficult, and should be a point of consideration in future studies.

Though small in number, several studies have attempted to address the feasibility of obtaining functional brain maps in individuals under sedation. It has previously been argued by Heinke and Schwarzbauer (2002) and Heinke et al. (2004) that anesthetic agents result in cerebral metabolic depression, potentially affecting brain responses to external stimulation. For example, the authors Heinke et al. (2004) reported dose-dependent effects of propofol on temporo-frontal regions involved in auditory language processing using passive fMRI in healthy volunteers, albeit these effects were most marked in frontal rather than temporal regions. Moreover, an fMRI study of controls by Davis et al. (2007) found that despite impairment of semantic and mnemonic processes at low levels of propofol sedation, perceptual processing of speech at even high levels of sedation is preserved, as characterized by engagement of temporal lobe regions previously implicated in receptive language processing.

The observations made in our study are consistent with the previous literature highlighting the utility of non-invasive techniques in identifying eloquent cortex among sedated patients (Papanicolaou et al., 2014). For example, using fMRI Souweidane et al. (1999) demonstrated consistent activation in left temporo-parietal and frontal regions in a group of eight children under propofol sedation, in response to passive auditory stimuli consisting of words and sentences, a pattern comparable to that derived in non-sedated subjects using a similar paradigm. Moreover, Gemma et al. (2009) compared the differential effects of propofol and midazolam on fMRI brain activation patterns during a passive listening task in 14 children, and reported that patients under propofol sedation exhibited primary auditory cortex activation patterns more similar to that observed in non-sedated adults. More recently, Lai et al. (2012) derived brain activation maps outlining cortical regions underlying speech and song perception in a group of low-functioning autistic children under light propofol sedation using passive auditory stimulation during fMRI. In addition, similar success was observed by Van Poppel et al. (2012) for the purpose of establishing hemispheric dominance for receptive language with MEG using a passive language mapping protocol in a series of 15 patients while under either sedation or during natural sleep (Stage I/II), three of whom exhibited no subsequent postoperative language deficits.

Given our criteria for determining hemispheric dominance in clinical practice, the findings from the present study also highlight the importance of the qualitative assessment of brain activation maps on an individual basis, as an adjunct to traditional quantitative estimates of laterality. Specifically, a large number of assessments based solely on the calculated LI, in either group of patients in the present study, were not concordant with judgments based on visual inspection of activation profiles associated with receptive language processing. As evidenced by the cases reviewed here, recognizing the degree to which regions supporting linguistic processes are engaged is equally, if not more, important for identifying the dominant hemisphere, than only taking into account differences in the amount of activity sources between the hemisphere. An example of this scenario, and one encountered numerous times in our clinical assessments, is the observation that despite a greater distribution (but lower absolute count) of activity sources within one hemisphere, a greater number of activity sources concentrated in one region (e.g., auditory cortex) of the other hemisphere results in an LI that may not accurately reflect the organization of receptive language in a given individual. Indeed, considering the potential for functional reorganization in neurological patients, especially in the case of individuals with space-occupying lesions, a more subjective approach to assessing laterality for receptive language may be warranted.

Collectively, the findings from our large-scale retrospective review constitute an indication for the efficiency of MEG in establishing hemispheric dominance for receptive language in children under sedation. Considering the challenges associated with assessing brain function in pediatric patients, the success of passive MEG in the context of the cases reviewed in this study support the utility of this method in pre-surgical language mapping. Future studies incorporating a multimodal imaging framework may further highlight the efficiency of non-invasive methods for functional mapping in challenging pediatric populations.

## Conflict of Interest Statement

The authors declare that the research was conducted in the absence of any commercial or financial relationships that could be construed as a potential conflict of interest.
